# Stochastic electrical stimulation of the thoracic or cervical regions with surface electrodes facilitates swallow in rats

**DOI:** 10.3389/fneur.2024.1390524

**Published:** 2024-07-09

**Authors:** In Kitamura, Michael Frazure, Kimberly Iceman, Takuji Koike, Teresa Pitts

**Affiliations:** ^1^Department of Mechanical and Intelligent Systems Engineering, The University of Electro-Communications, Chōfu, Tokyo, Japan; ^2^Department of Physiology, School of Medicine, University of Louisville, Louisville, KY, United States; ^3^Department of Speech, Language, and Hearing Sciences and Dalton Cardiovascular Center, University of Missouri, Columbia, MO, United States

**Keywords:** swallow, dysphagia, spinal cord stimulation, central pattern generator, rehabilitation

## Abstract

**Introduction:**

Aspiration pneumonia, a leading cause of mortality, poses an urgent challenge in contemporary society. Neuromuscular electrical stimulation (NMES) has been commonly used in dysphagia rehabilitation. However, given that NMES at motor threshold targets only specific muscles, it carries a potential risk of further compromising functions related to swallowing, respiration, and airway protection. Considering that the swallow motor pattern is orchestrated by the entire swallow pattern generator (the neural mechanism governing a sequence of swallow actions), a rehabilitation approach that centrally facilitates the entire circuit through sensory nerve stimulation is desirable. In this context, we propose a novel stimulation method using surface electrodes placed on the back to promote swallowing.

**Methods:**

The efficacy of the proposed method in promoting swallowing was evaluated by electrically stimulating sensory nerves in the back or neck. Probabilistic stimulus was applied to either the back or neck of male and female rats. Swallows were evoked by an oral water stimulus, and electromyographic (EMG) activity of the mylohyoid, thyroarytenoid, and thyropharyngeus muscles served as the primary outcome measure.

**Results:**

Gaussian frequency stimulation applied to the skin surface of the thoracic back elicited significant increases in EMG amplitude of all three swallow-related muscles. Neck stimulation elicited a significant increase in EMG amplitude of the thyroarytenoid during swallow, but not the mylohyoid or thyropharyngeus muscles.

**Discussion:**

While the targeted thoracic spinal segments T9-T10 have been investigated for enhancing respiration, the promotion of swallowing through back stimulation has not been previously studied. Furthermore, this study introduces a new probabilistic stimulus based on Gaussian distribution. Probabilistic stimuli have been reported to excel in nerve stimulation in previous research. The results demonstrate that back stimulation effectively facilitated swallow more than neck stimulation and suggest potential applications for swallowing rehabilitation.

## 1 Introduction

Dysphagia refers to the difficulty in safely forming or transferring food boluses from the oral cavity to the esophagus. Untreated dysphagia can lead to the aspiration of food particles and saliva containing bacteria into the lungs, resulting in inflammation and infection. Aspiration pneumonia constitutes almost 70% of pneumonia cases (1), ranking as the third leading cause of death among elderly individuals in Japan (2). Additionally, the prevalence of dysphagia among the elderly has been reported as high as 33% in the United States (3), 23% in Europe (4), 33% in individuals aged 80 and above in Europe (4), 14% in Japan (5), and 34% in South Korea (6). Therefore, the development of rehabilitation techniques for dysphagia is of utmost urgency in contemporary aging societies.

An established rehabilitation technique for dysphagia is neuromuscular electrical stimulation (NMES). This often involves stimulating the muscles in the anterior neck region through surface electrodes. However, this therapy does not directly induce swallow and is limited in its effectiveness as it primarily targets specific muscles rather than facilitation of the central swallow pattern generator. Moreover, there are concerns that electrical stimulation therapy may lead to rapid muscle fatigue, potentially further compromising functions related to swallow, respiration, and airway protection with stimulation (7). Since the sequence of swallow muscle activities is not solely dependent on specific muscles but also on the activation of the entire swallow reflex circuit, it is logical to explore rehabilitation methods to activate the entire swallow reflex circuit through sensory nerve system stimulation (8).

As a method to activate sensory nerves, it has been proposed that dysphagia can be treated by directly applying magnetic stimulation to the areas near the primary somatosensory cortex, as a large portion of the primary somatosensory cortex is associated with swallow (9–12). While this approach holds promise for improving dysphagia, there are challenges associated with the enlargement of stimulation devices, resulting in high implementation costs for rehabilitation equipment (13–15).

Respiration and swallow are closely related, with both behaviors relying on signals from various sensory nerves. Generally, the respiratory cycle can be divided into inspiration, early expiration (also known as post-inspiration), and late expiration phases. Swallow is inhibited during the inspiration phase (16, 17). Based on this fact and considering the desirability of the expiration phase for swallow timing, we proposed a method that involves electrical stimulation of sensory nerves which travel through pathways within the spinal cord. We used Gaussian frequency stimulation applied through surface electrodes placed either near the thoracic spine T9-T10 on the dorsal side, or on the anterior lateral region of the neck to test the hypothesis that this novel stimulus would facilitate swallow.

## 2 Materials and methods

### 2.1 Animals

The experiments were conducted using spontaneously breathing retired breeder Sprague Dawley rats [*N* = 16, 8 male (0.63 ± 0.11 kg), 8 female (0.26 ± 0.02 kg); 9–12 months of age]. Ethical approval for the protocol was obtained from the Institutional Animal Care and Use Committee (IACUC) at the University of Louisville.

The animals were initially anesthetized through inhalation of gaseous isoflurane (1.5–2%). Subsequently, pentobarbital sodium was administered at a dosage of 25 mg/kg via a femoral vein catheter (IV). Thereafter, isoflurane was ceased, and supplementary doses of pentobarbital sodium (ranging from 1 to 4 mg/kg via IV injection) were administered. To reduce tracheal secretions, atropine sulfate was administered at the beginning of the experiment at a dose of 0.01 mg/kg via IV injection. The depth of anesthesia was assessed every 15 min by evaluating withdrawal reflexes, blinking, and jaw tone. At the same intervals, body temperature, respiratory rate and heart rate were monitored. If the anesthetic level was deemed insufficient based on these evaluations, supplemental doses of pentobarbital sodium (ranging from 1 to 4 mg/kg via IV injection) were administered as required (18). Prior to initiating the stimulation protocol, anesthetic depth was confirmed, and then no additional doses were administered during the experiment. Body temperature was maintained using a heating pad.

### 2.2 Electrophysiology recording

**Figure 1:**
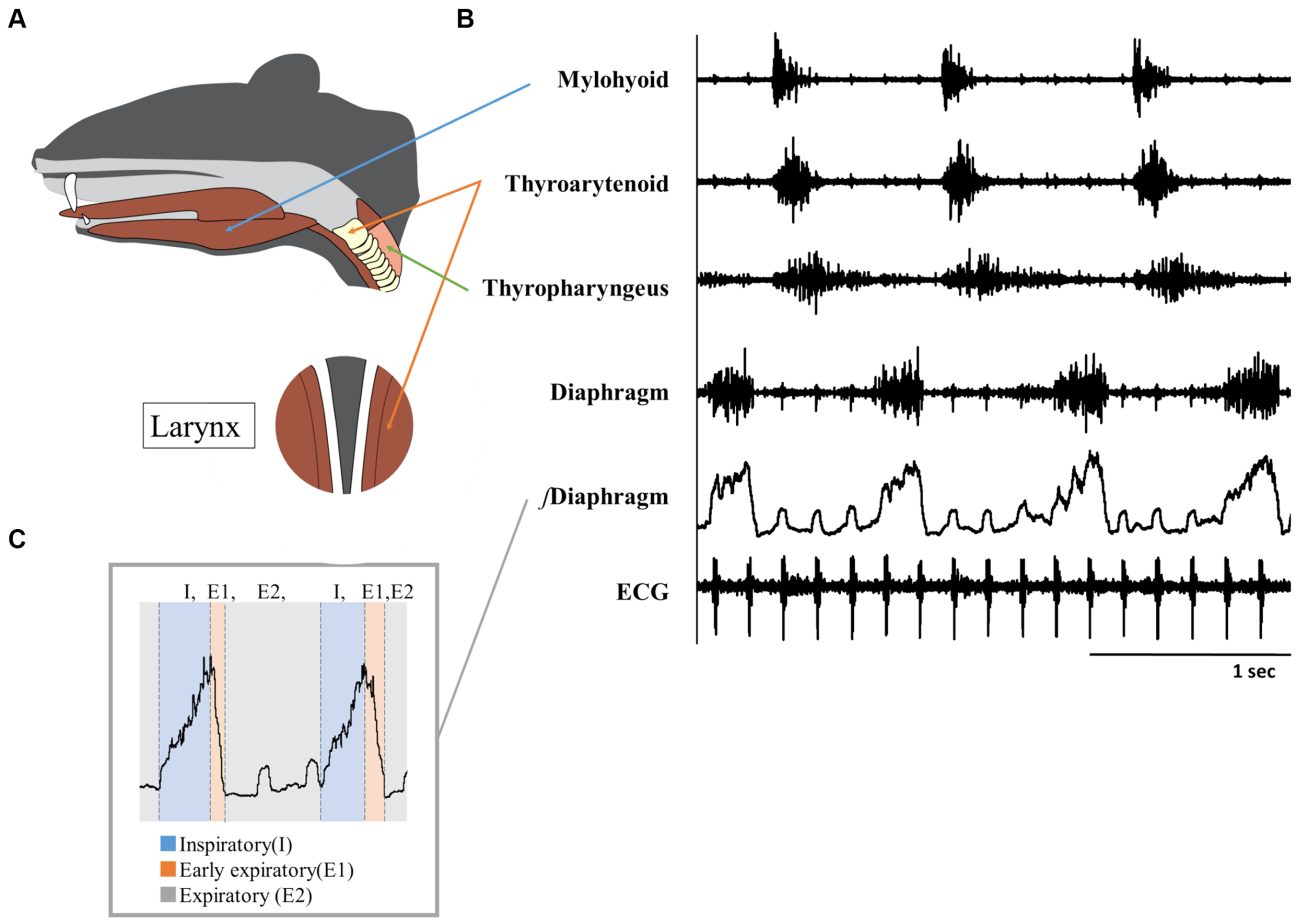
**(A)** Diagram illustrating the anatomical positions of the muscles targeted for EMG recording, **(B)** Representative EMG trace during swallows, **(C)** Methodology for segmenting respiratory phases using diaphragm EMG.

Electromyographic (EMG) recordings of all muscle activities were obtained using bipolar insulated fine-wire electrodes (AM Systems stainless steel No. 791050) placed on each muscle following the technique described by Basmajian and Stecko (19). Three swallow muscles were evaluated: mylohyoid, thyroarytenoid, and thyropharyngeus muscles. The costal diaphragm was recorded to evaluate breathing. Figure 1 illustrates the anatomical locations of each muscle and typical traces of muscle activity during pharyngeal swallow.

The recording electrodes were surgically placed as follows: The mylohyoid muscle electrodes were inserted into both the right and left mylohyoid muscles after making a small horizontal incision along the midline of the chin to expose the surface of the digastric muscle. The thyroarytenoid muscle electrodes were inserted anteriorly through the window of the cricothyroid membrane, reaching the front of the vocal cords. For the thyropharyngeus muscle, the electrodes were inserted into the posterior part of the thyropharyngeus muscle by curving the insertion needle. The diaphragm electrode was positioned by inserting the injection needle caudally, guided by palpating and elevating the xiphoid process. Electrocardiogram (ECG) activity was recorded by placing electrodes on the left pectoralis major and right caudal gastrocnemius muscles. ECG was used for measuring heart rate and removing cardiac artifacts from the EMG traces.

### 2.3 Experimental protocol

**Figure 2:**
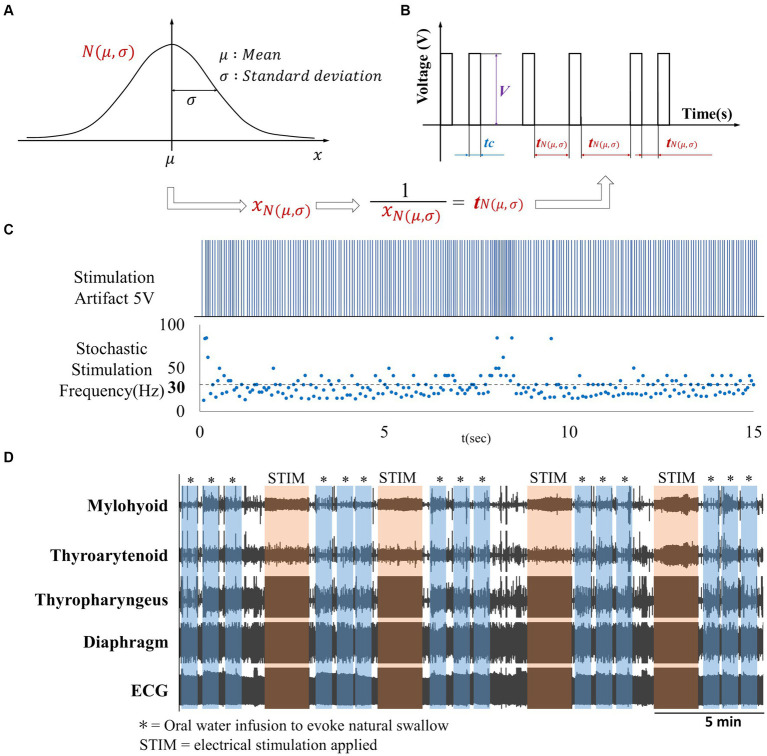
**(A)** Gaussian distribution, **(B)** Gaussian frequency stimulation waveform. Waveform with intermittent single-pulse stimulation is applied based on the reciprocal of the frequency $x_{N\left(\mu ,\sigma \right)}$ randomly generated according to the Gaussian distribution depicted in **(A,C)** Artifact produced by Gaussian frequency electrical stimulation with a mean frequency of 30 Hz, **(D)** Overview of EMG activity throughout the experiment.

**Figure 3:**
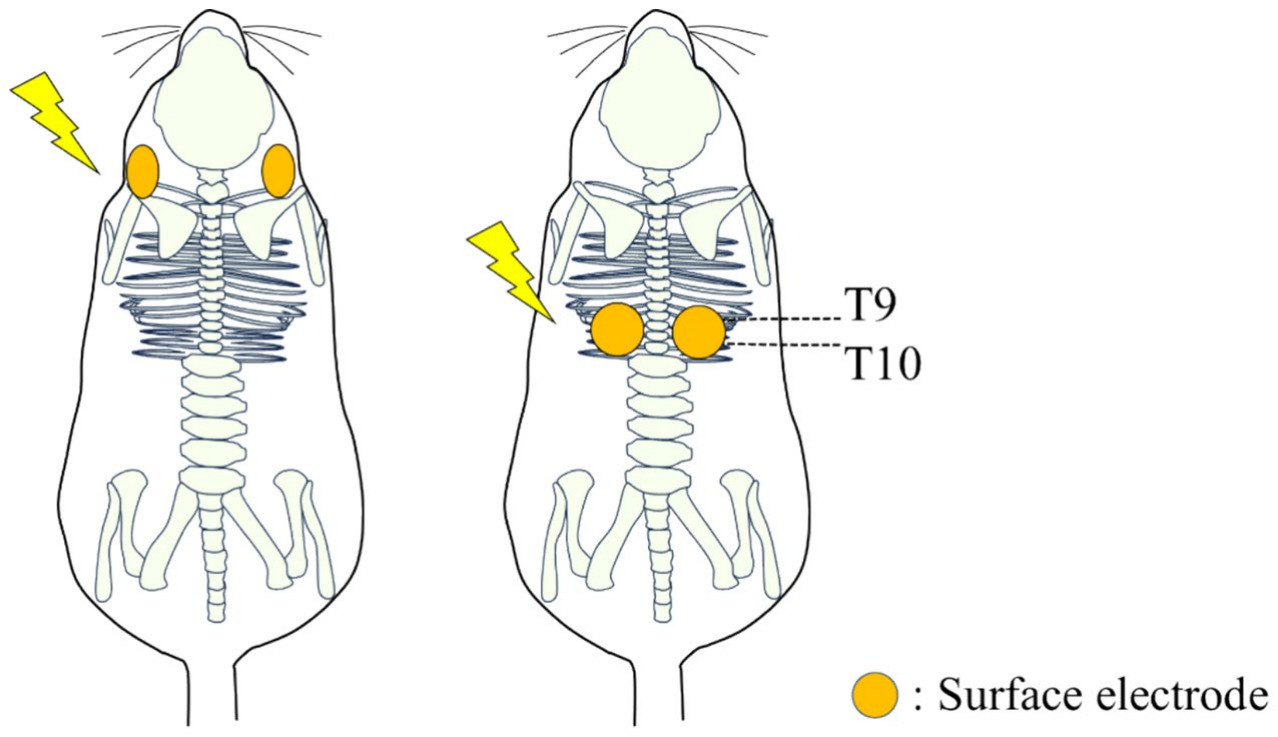
Diagram illustrating the placement of wet electrodes for electrical stimulation on the neck and back of rats.

Our objective was to investigate the effects on swallow of electrical stimulation via surface electrodes placed on the back (T9-T10) or neck (Lateral side of the neck) regions. In this study, we introduce a novel probabilistic stimulus based on Gaussian distribution (Figure 2B). In the figure, $t_{c}$ denotes a pulse width, and V denotes a voltage of the pulse waveform. Let $N\left(\mu ,\sigma \right)$ be a Gaussian distribution with mean μ and standard deviation σ (Figure 2A). For each pulse, the value of the interval between pulses are set as $t_{N\left(\mu ,\sigma \right)}$ , which is the inverse of the value $x_{N\left(\mu ,\sigma \right)}$ realized value from $N\left(\mu ,\sigma \right)$ . The electrical stimulator used in this experiment was the Grass Stimulator Model S88 (RRID:SCR_016192) (Grass, Inc., Warwick, RI). The waveform generation program was implemented using custom scripts in Spike2 software (CED, Cambridge Electronic Design). In this experiment, the parameters for the stimulation waveform were set with a pulse width of 15 ms and an output voltage of 5 V. Surface electrodes were placed as follows: in the back stimulation group, surface electrodes were positioned on the dorsal thoracic area (T9-T10) of 8 rats [4 males (0.64 ± 0.14 kg) and 4 females (0.26 ± 0.02 kg)]; in the neck stimulation group, surface electrodes were positioned bilaterally near the larynx on the cervical area of 8 rats [4 males (0.61 ± 0.06 kg) and 4 females (0.26 ± 0.02 kg)] (Figure 3). Surface electrodes with a diameter of 25 mm were used for female rats, while electrodes with a diameter of 30 mm were used for male rats. The electrodes were fixed in place with tape after shaving the hair on the surface of the application site. Using a probabilistic paradigm, pulses were applied at each site for 120 s in four repetitions (designated as S1-S4) with a frequency randomly generated according to the Gaussian distribution set with a mean of 30 Hz and a standard deviation of 10 Hz (Figure 2C). Previous studies on spinal stimulation in rats have demonstrated that muscle activity can be induced with low current intensities of approximately 1 mA (20, 21). The current applied in this study ranged from 0.75 mA to 1.1 mA, which was lower than the current threshold (14 mA) required to induce muscle contraction (22). It has been found that when high-amplitude stimulation is used peripherally, exceeding the optimal amplitude may block orthodromic action potentials generated within the spinal cord (23).

We recorded EMG activity from three swallow-related muscles (mylohyoid muscle, thyroarytenoid muscle, thyropharyngeus muscle), as well as the diaphragm, along with heart rate and respiratory rate. Oral water administration was performed by injecting 1 cc of room temperature water into the oropharynx through a thin polyethylene catheter (0.5–1.0 mm outer diameter, 0.5 inches in length) placed at the base of the tongue. Three oral water administrations were conducted immediately before stimulation (control) and immediately after each of the four stimulation trials (S1-S4) (Figure 2D). At the conclusion of the experimental protocol, euthanasia was induced through an overdose of pentobarbital sodium followed by intravenous administration of 1 cc of saturated potassium chloride. Pneumothorax was also induced as a secondary euthanasia method.

### 2.4 Analysis

The EMG signals were amplified using Grass P511 AC Amplifiers (Natus Neurology), bandpass-filtered (200–5,000 Hz), recorded at a sampling rate of 10 KHz (1,401 Power3 + ADC16 Expansion, Cambridge Electronic Design), and analysed using Spike2 Software (RRID:SCR_000903) (v8, Cambridge Electronic Design). EMG signals were rectified and integrated with a time constant of 20 ms. Swallows were identified by coordinated bursts in EMG activity of the mylohyoid, thyroarytenoid, and thyropharyngeus muscles. Swallow events were included if they were induced within 30 s after oral water infusion. To normalize EMG values across animals, the percent change was calculated by dividing the average maximum EMG amplitude during post-stimulation swallows (S1-S4) by the average maximum EMG amplitude during control pre-stimulation swallows.

Changes in respiration were evaluated by determining the phase based on the diaphragmatic EMG. Inspiration (I) was defined as the period from the onset of diaphragmatic activity to the peak of diaphragmatic burst. Expiration (E) was defined as the period from the peak of diaphragmatic activity to the onset of the subsequent diaphragmatic activation. Furthermore, expiration was subdivided into early expiration (E1; from the peak of diaphragmatic amplitude to diaphragmatic quiescence) and late expiration (E2; from the offset of diaphragmatic activity to the onset of the subsequent diaphragmatic activity) (24) (Figure 1C). To assess the variations in the three phases (I, E1, E2) within a single breath, we conducted comparisons to normalize by dividing the time duration of each phase by the total duration of one respiratory cycle. This approach enables the elimination of differences in respiratory cycles between measurement trials, allowing for the observation of changes in the proportion of each respiratory phase. Swallow duration was defined as the interval from the onset of activity in the mylohyoid muscle to the cessation of thyropharyngeus muscle activity.

All measures are reported as mean ± standard deviation (SD). The following parameters were measured and compared between the swallows that were induced in the pre-stimulus (control) and post-stimulus (S1-S4) time periods: amplitudes of the three swallow-related muscles, diaphragm amplitude, duration of each swallow, respiratory rate per minute, heart rate per minute, and proportion of each respiratory phase within one respiratory cycle. Statistical analysis was performed using Prism (GraphPad) software. Normality tests were conducted, and then either analysis of variance (ANOVA) or Friedman tests were performed as appropriate to test for main effects. *Post hoc* multiple comparison tests were performed (Dunnet’s for one-way parametric, Fisher’s LSD for two-way parametric, and Dunn’s for non-parametric) to assess specific differences between the control and stimulus groups. Differences with *p*-values below 0.05 were considered statistically significant.

## 3 Results

**Figure 4:**
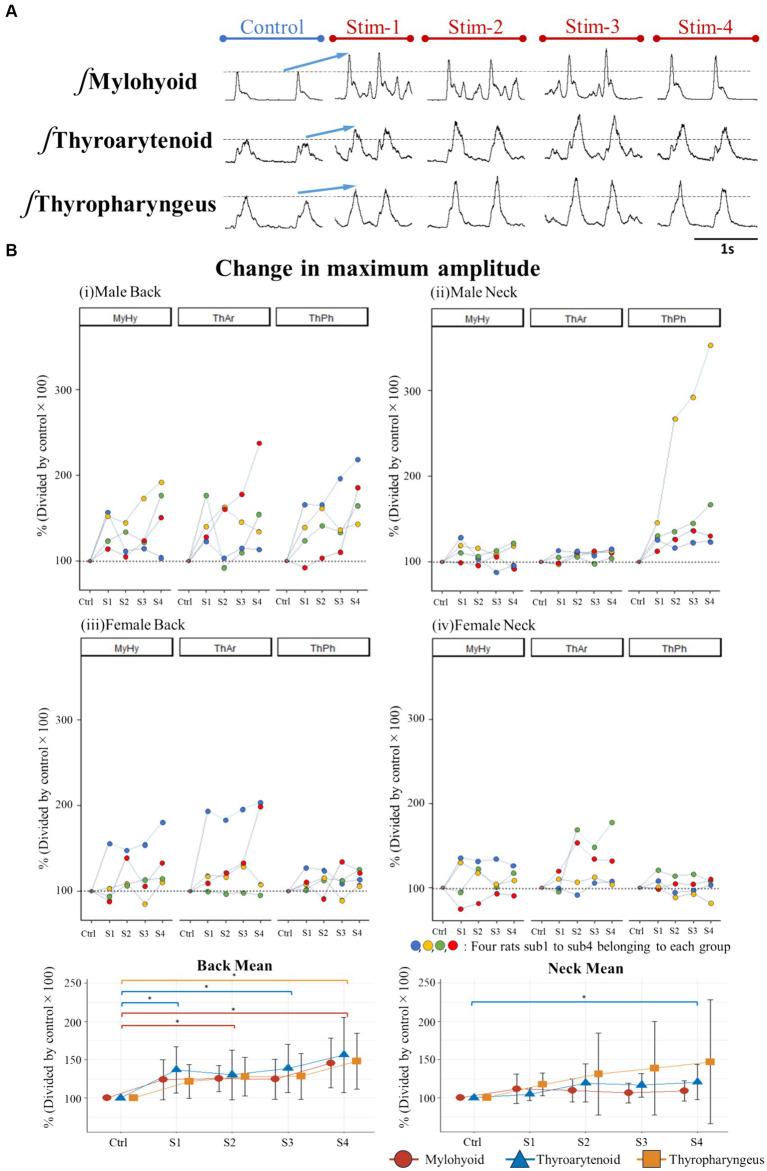
**(A)** Example of changes in EMG amplitude of swallow muscles when stimulating the back of male rats, **(B)** Percent change in maximum amplitude of swallow EMG potentials immediately after stimulation (S1-S4) relative to control (set as 100%) for each rat and for the back and neck group means.

First, we evaluated changes in maximum EMG amplitude of three swallow-related muscles (mylohyoid, thyroarytenoid, and thyropharyngeus) (Figure 4B) before and after the application of Gaussian frequency electrical stimulation (four stimulation trials each, labelled S1-S4). The stimulating electrodes were placed bilaterally on the surface of the skin either at the posterior T9-T10 thoracic spine region, or at the anterior cervical region bilateral to the larynx. We compared EMG amplitudes during swallow induced by oral water infusion before stimulation (control) and immediately after each electrical stimulation (S1 to S4). For the back stimulation groups (T9-T10 region), ANOVA detected a significant effect of stimulus on EMG amplitude of the mylohyoid [*F*_(4,28)_ = 6.219, *p* = 0.006], thyroarytenoid [F_(4,28)_ = 4.798, *p* = 0.02], and thyropharyngeus [F_(4,28)_ = 5.909, *p* = 0.009] muscles during swallow (Figure 4A). *Post hoc* comparisons showed that mylohyoid EMG amplitude increased following the second (*p* = 0.02) and fourth (*p* = 0.02) trials of back stimulation. Thyroarytenoid EMG amplitude increased following the first (*p* = 0.005) and third (*p* = 0.04) trials of back stimulation. Thyropharyngeus EMG amplitude increased following the fourth (*p* = 0.04) trial of back stimulation. For the anterior neck stimulation groups, ANOVA detected a significant effect of stimulus on EMG amplitude of the thyroarytenoid [χ^2^(5) = 10.70, *p* = 0.03], but not the mylohyoid or thyropharyngeus muscles during swallow. *Post hoc* comparisons showed that thyroarytenoid EMG amplitude increased following the fourth (*p* = 0.02) trial of neck stimulation. An ANOVA omnibus test detected a significant interaction effect between sex and stimulus treatment [*F*_(4,24)_ = 3.19, *p* = 0.03] and a significant effect of sex [*F*_(1,6)_ = 6.92, *p* = 0.04] for the thyropharyngeus EMG amplitude following back stimulation.

**Figure 5:**
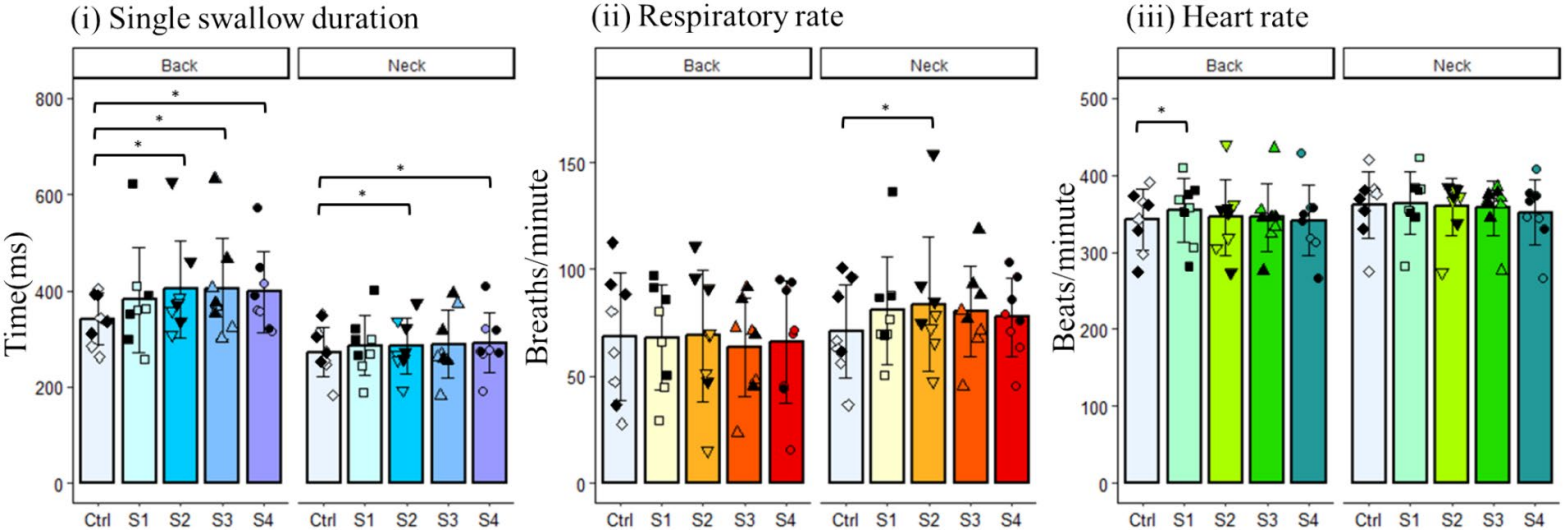
Changes in various physiological parameters of the back and neck groups. Filled black symbols represent male data points; open symbols represent female data points.

We then evaluated the effects of the back and neck Gaussian frequency electrical stimulation on swallow duration, respiratory rate, and heart rate (Figure 5). Swallow duration increased in the back stimulation group [χ^2^(5) = 12.50, *p* = 0.01], and *post hoc* comparisons showed a significant effect following the second (*p* = 0.03), third (*p* = 0.045), and fourth (*p* = 0.01) back stimulus trials. While omnibus ANOVA did not detect an overall difference in swallow duration for the neck stimulation group [*F*_(4,28)_ = 2.177, *p* = 0.2], *post hoc* tests detected a significant increase in swallow duration following the second (*p* = 0.009) and fourth (*p* = 0.02) neck stimulus trials (Figure 5i). There were no differences detected for the effect of back stimulation on respiratory rate. However, respiratory rate was increased following neck stimulation [*F*_(4,28)_ = 4.113, *p* = 0.04], and *post hoc* tests detected a significant increase in respiratory rate following the third (*p* = 0.03) neck stimulus trial (Figure 5ii). While omnibus ANOVA did not detect an overall difference in heart rate for the back stimulation group [F_(4,28)_ = 0.925, *p* = 0.4], *post hoc* tests detected a significant increase in heart rate following the first (*p* = 0.01) back stimulus trial. There were no differences detected for the effect of neck stimulation on heart rate (Figure 5iii).

**Figure 6:**
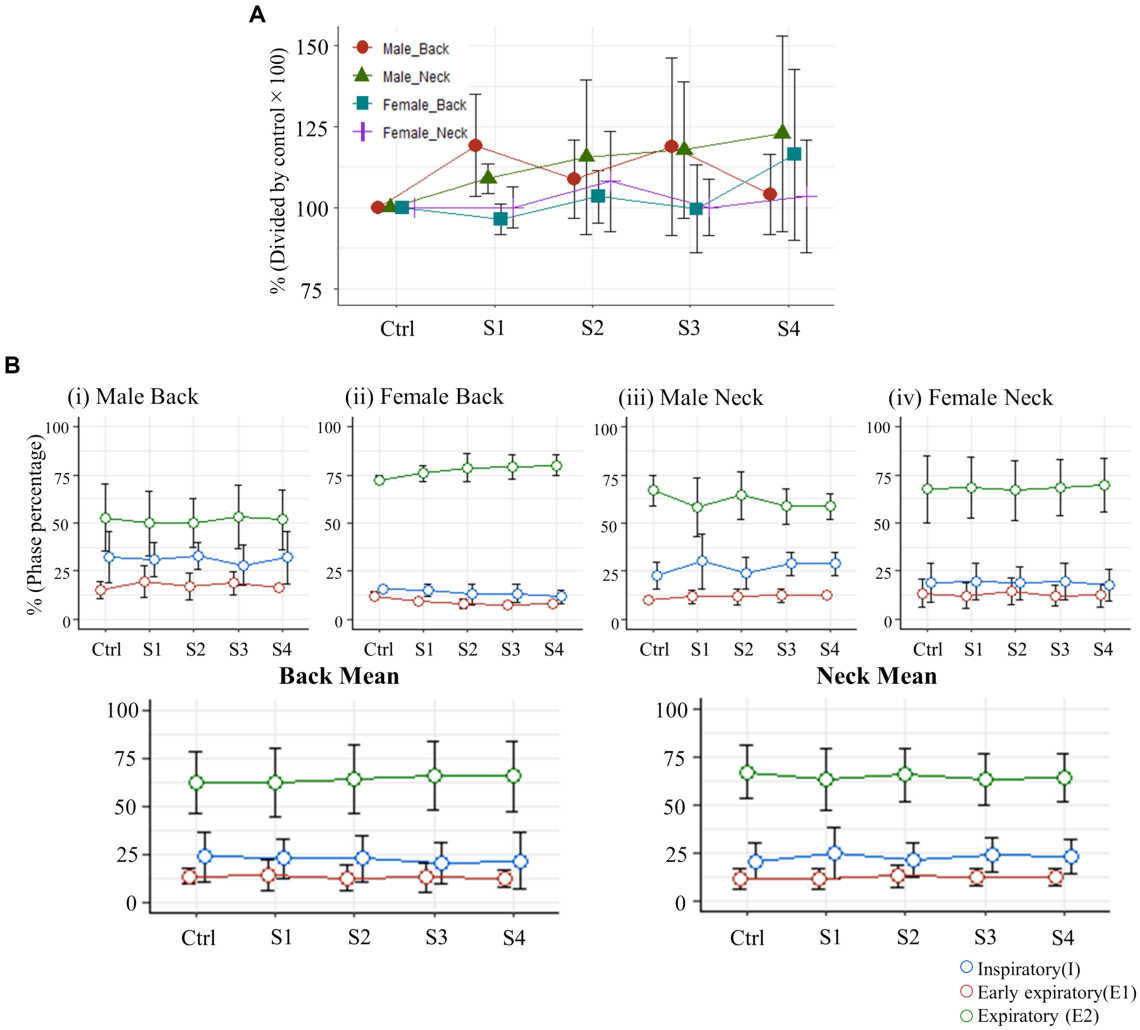
**(A)** Changes in the maximum amplitude of diaphragm muscle EMG activity (S1-S4) immediately after each stimulation trial, expressed as a percent change relative to the control diaphragm EMG activity (normalized to 100%). **(B)** Variations in the proportion of each respiratory phase within one respiratory cycle, expressed as a percentage.

Finally, we assessed whether electrical stimulation at the neck or back affects diaphragm amplitude during breathing or modulates respiratory phase. There were no significant changes in the maximum EMG amplitude of the diaphragm for the back or neck stimulus groups (Figure 6A). Omnibus F-tests conducted for each group revealed no significant changes in the proportions of the I (inspiratory), E1 (early expiratory), and E2 (expiratory) phases of respiration, and post-hoc multiple comparisons similarly indicated no significant differences in any of the groups (Figure 6B).

## 4 Discussion

This study aimed to investigate whether a novel Gaussian frequency stimulation paradigm applied to skin of the back could facilitate swallow. Gaussian frequency stimulation applied to the skin surface of the thoracic back (at the T9-T10 level) elicited significant increases in EMG amplitude of all three swallow-related muscles at a variety of timepoints (S1-S4), indicating that this stimulation method facilitates swallow. This effect on thyropharyngeus EMG activity was more pronounced in the male group compared to the female group. Swallow duration was also increased after stimulation of the back or the neck.

Swallow preferentially occurs in the expiratory phase of breathing. While swallow occurs during post-inspiration in rats and mice (25, 26), it is most likely to occur later in expiration in cats and humans (27–29). While swallow is mediated by cranial nerves, it is impaired by spinal cord injury (18), and is influenced by afferent feedback from pulmonary stretch receptors that are activated during inspiration (16, 25). Activation of spinal afferents in the chest wall also affects swallow (25), and rib vibration increases laryngeal tone, which is important during respiration and for airway protection during swallow (30). Swallow also influences the phase coordination of the respiratory cycle, including inspiration and expiration (16, 31, 32). Indeed, the swallow and respiratory pattern generators are both situated in the brainstem, adjacent to each other and overlapping at least somewhat.

Spinal pathways are also involved in respiration. The load compensation reflex that occurs during airway occlusion is spinally mediated (33), and stimulus of the chest wall by vibration or electrical stimulation induces significant changes to respiration (34–41). The muscles that we recorded are representative of key movements that occur during the pharyngeal phase of swallow. The mylohyoid represents laryngeal elevation, the thyroarytenoid represents laryngeal adduction and the thyropharyngeus represents pharyngeal constriction. Each of these components is necessary for airway protection and bolus passage through the pharynx and into the esophagus during swallow. Each muscle is innervated by different nerves and/or branches, suggesting that a stimulus which would affect all these muscles would act centrally. Sensory nerves involved in respiration or swallowing are likely to influence both functions. The precise mechanisms by which spinal afferent information influences the swallow and respiratory central pattern generators, differentially or in concert, to produce changes in swallow and breathing remain to be discovered.

The Gaussian frequency stimulation used in this study differs from the conventional fixed-frequency single-pulse waves commonly used in therapeutic devices. Applying stimulation with Gaussian frequency can enhance the detection of weak signals in sensory signal transmission (42), and varying the applied frequency is expected to prevent adaptation to the stimulation (43). NMES using commercially available hardware stimulate with a fixed frequency of 70 Hz (44), investigations using *in vivo* animal models traditionally use fixed frequency stimulation between 5 and 30 Hz. Stimulation of the SLN with 20–30 Hz fixed frequency evokes rapid continuous swallow with short latencies and low threshold (45–49). Recent research has reported that probabilistic frequency stimulation led to a more significant swallow response compared to fixed-frequency stimulation of the superior laryngeal nerve (SLN) (50). In that study, stochastic SLN stimulation decreased swallow latency when compared to fixed-frequency stimulation, and increased swallow number and swallow EMG amplitudes; thus it appeared to increase excitability of the swallow pattern generator. Some of these same effects are observed when the frequency is increased in the setting of SLN stimulation with a fixed frequency (51). Adding stochastic variance to the electrical stimulation signal may work similarly to increasing the fixed frequency, while maintaining the overall stimulation delivered. Furthermore, since the swallow-related muscle groups are not directly stimulated to contract during swallow, the method we used in this study importantly does not act as an inhibitory factor during natural swallow.

The therapeutic effects of spinal cord stimulation have been demonstrated in various medical conditions, improving sensory-motor function and autonomic nervous system functions (52, 53). Two approaches for spinal cord stimulation are epidural spinal cord stimulation (eSCS), which requires surgical intervention, and non-invasive stimulation techniques, such as magnetic or electrical stimulation applied to the skin surface over the spinal cord. In epidural SCS, studies have explored the selective stimulation of different segments of the cervical, thoracic, and lumbar spine to treat disorders related to cardiorespiratory, motor function, and excretion (54–56). For instance, stimulation at the C4 level in a rat model with spinal cord injury at C3-C5 improved normal breathing and blood oxygen concentration, induced diaphragmatic nerve response time, and produced frequency-dependent changes (short-term facilitation) (57). Furthermore, in the case of patients with chronic paralysis, voluntary leg movements recovery has been observed through electrical stimulation by implanting electrodes at the T11-T12 level (58).

In previous research on transcutaneous electrical stimulation using surface electrodes, electrical stimulation near the thoracic spine (T11-L1) with surface electrodes induced an increase in sensation and contraction pressure in the anal-rectal region in patients with neurogenic bowel dysfunction following spinal cord injury (59). Additionally, for individuals with idiopathic lower urinary tract dysfunction after spinal cord injury, high-frequency biphasic burst wave transcutaneous spinal cord electrical stimulation at T11 and L1 resulted in immediate effects such as decreased overactivity of voiding muscles and improved incontinence (60).

While research on the effects of spinal cord stimulation on swallow movements remains limited, significant progress has been made in studying how spinal cord stimulation influences respiratory regulation. DiMarco and colleagues demonstrated that stimulation at the thoracic spine levels T9-T11 facilitates laryngeal and abdominal expiratory muscle activity (41, 61–63). In one study, patients with cervical C5-C6 injuries who were unable to cough normally had electrodes implanted and stimulation was applied to either T9, T11, or L1 of the thoracic spine, either individually or in combination. The results showed an increase in airway pressure and expiratory flow, allowing patients to induce coughing (62). Other areas of the thoracic spine are recognized as sites to modify ongoing inspiratory drive (64–66). In another study on spinal cord stimulation in dogs, stimulating at T2-T3 allowed for the maintenance of 6 h of respiration. Analysis of inspiratory muscles revealed firing frequencies of motor units equivalent to spontaneous breathing, leading to the activation of both the diaphragm and inspiratory intercostal muscles (64). In contrast, in the current study, stimulation of the T9-T10 region on the back did not produce significant differences in any respiratory phases. The disparity in these results is believed to stem from the fact that, in prior studies, high-intensity direct stimulation of the spinal cord (T9-T10) was employed, rather than the transcutaneous low-intensity stimulation via surface electrodes conducted in this study. This distinction implies a significant difference in the actual current flowing through the spinal cord. It is best if any therapeutic stimulation targeting swallow does not affect respiration, and it is plausible that higher-intensity stimulation than what was performed in the current protocol is necessary to induce the off-target effects on expiration. However, because we calculated respiratory parameters directly after, rather than during, the electrical stimulation, changes in airway pressure and respiratory muscle activity during stimulation as reported in prior studies cannot be discounted. In addition, in contrast to the experimental model of spinal cord injury utilized in prior research, the present study employed a healthy rat model. Prior to experimentation, stable spontaneous respiration was confirmed, and no decline in respiratory capacity was observed.

Possible therapeutic applications for Gaussian electrical stimulation include stabilizing breathing (67, 68) and infant suck-swallow patterns (69), and decreasing tremor and bradykinesia using deep brain stimulation in patients with Parkinson’s disease (70). In clinical dysphagia therapy, an 80 Hz fixed frequency has been successfully used (71) in the short term (7, 72). However, in several studies long-term therapeutic use has not been successful (73–77).

In the present study we found that surface probabilistic electrical stimulation of sensory nerves in the back or neck of rats can enhance activation of certain muscles during swallow in rats immediately after stimulation. Probabilistic electrical stimulation should be explored further in future experiments to test the sustained effectiveness of facilitating swallow over longer time courses. Previous studies have shown long-term brain plasticity after sensory nerve stimulation (78). Additionally, although the stimulation waveform in this study differs, experiments using transcranial random noise stimulation (tRNS) have suggested the rapid modulation of voltage-dependent sodium channels (79), and the application of tRNS to the pharyngeal cortex has demonstrated a sustained two-hour increase in pharyngeal motor-evoked potentials (80). In conclusion, the results from the current study suggest that stochastic electrical surface stimulation, especially to the thoracic T9-T10 region, may show promise for improving swallow dysfunction. This stimulation method should be explored for therapeutic potential in further studies.

## Data availability statement

The raw data supporting the conclusions of this article will be made available by the authors, without undue reservation.

## Ethics statement

The animal studies were approved by Institutional Animal Care and Use Committee (IACUC) at the University of Louisville. The studies were conducted in accordance with the local legislation and institutional requirements.

## Author contributions

IK: Conceptualization, Data curation, Formal analysis, Investigation, Methodology, Project administration, Resources, Software, Supervision, Validation, Visualization, Writing – original draft, Writing – review & editing. MF: Conceptualization, Data curation, Investigation, Methodology, Project administration, Resources, Supervision, Validation, Writing – review & editing. KI: Conceptualization, Formal analysis, Investigation, Supervision, Validation, Writing – review & editing. TK: Supervision, Writing – review & editing. TP: Conceptualization, Data curation, Formal analysis, Funding acquisition, Investigation, Methodology, Project administration, Resources, Supervision, Validation, Writing – review & editing.
